# Rapid Detection of Six Glucocorticoids Added Illegally to Dietary Supplements by Combining TLC with Spot-Concentrated Raman Scattering

**DOI:** 10.3390/molecules23071504

**Published:** 2018-06-21

**Authors:** Li Li, Xin Liang, Tao Xu, Feng Xu, Wei Dong

**Affiliations:** School of Pharmacy, Qiqihar Medical University, Qiqihar 161006, China; lilianlinsuo@163.com (L.L.); Xutao@qmu.edu.cn (T.X.); 15845205504@163.com (F.X.); pingguoweiweiwei@126.com (W.D.)

**Keywords:** thin layer chromatography (TLC), spot concentrated Raman scattering (SCRS), glucocorticoids, dietary supplement, illegally adulterants

## Abstract

The objective of this study was to establish a novel method for rapid detection of six glucocorticoids (prednisone, prednisone acetate, prednisolone, hydrocortisone, hydrocortisone acetate, and dexamethasone) added illegally in dietary supplements simultaneously by combining thin layer chromatography (TLC) with spot-concentrated Raman scattering (SCRS). The doping ingredients were separated by TLC, and viewed and located with UV light (254 nm), enriched by chromatography, then Raman spectra were directly detected by a Raman Imagine microscope with 780 nm laser source. This method had complementary advantages of TLC and Raman spectroscopy, which enhanced the specificity of the test results. The limit of detection (LOD) of the reference substances were 4 μg, 4 μg, 4 μg, 6 μg, 6 μg, and 4 μg, respectively. The method was used to study the six glucocorticoids added illegally in five dietary supplements. Fake drugs had been detected. The study showed that the TLC-SCRS method is simple, rapid, specific, sensitive, and reliable. The method could be used for effective separation and detection of six chemical components used in dietary supplement products, and would have good prospects for on-site qualitative screening of dietary supplement products for adulterants.

## 1. Introduction

A dietary supplement is a commercially available product that is consumed as an addition to the usual diet and includes vitamins, minerals, herbs (botanicals), amino acids, and a variety of other products. Marketing claims for some dietary substances include improvements in overall health status, enhancement of cognitive or physical performance, increase in energy, loss of excess weight, attenuation of pain, and other favorable effects [1]. Recently, the demand for dietary supplements is increasing significantly due to the belief that the dietary supplement materials are harmless and have fewer side effects than chemical drugs [2]. Many studies have reported on the adulteration of dietary supplements with synthetic drugs [3,4,5,6]. The chemical substances that are most likely added in dietary supplements are glucocorticoids. To seize better effects, some producers added chemical drugs, such as prednisone, prednisone acetate, prednisolone, hydrocortisone, hydrocortisone acetate, and dexamethasone illegally [7,8,9,10], posing a severe threat to public health. However, abuse of glucocorticoids in dietary supplements results in an unpredictable risk of illness due to side effects, including the possibility of poor control of the adrenal axis, opportunistic infection, diabetes mellitus, osteoporosis [11], Cushing’s syndrome, pneumonia, asthma, and herpeskeratitis [12,13].

Various analytical techniques used for reliable, high-quality analyses of adulterants in dietary supplements have been developed, including high-performance liquid chromatography-mass spectrometry (HPLC-MS) [14], gas chromatography-mass spectrometry (GC-MS) [15,16], and high-performance liquid chromatography (HPLC) with ultraviolet-visible detection (UV–VIS) [17,18]. However, the disadvantages of all the methods above include being of high cost, time-consuming, and complicated. Thus, in our study, we chose a high-speed, high-selectivity, low-cost screening method, which combined thin layer chromatography (TLC) with spot-concentrated Raman scattering (SCRS) to directly identify trace adulterants. Raman spectroscopy is not a separation technique, so we could not use it to differentiate several components in a mixture. Among many separation methods, TLC is relatively simple and cheap [19,20,21]. Compared to other TLC methods, including TLC-dynamic surface enhanced Raman spectroscopy (DSERS) [22,23,24,25] and TLC-surface enhanced Raman spectroscopy (SERS), the new method in our study needs no reference substance. The combination of TLC and SCRS is suitable for rapid detection of health supervision when portable Raman spectroscopy is used, due to its simplicity, rapidity, and specificity.

The TLC-SCRS (combining thin layer chromatography with spot-concentrated Raman scattering) method established in this article was successfully applied for rapid on-site detection of six glucocorticoids which adulterated dietary supplements. In this article, a great challenge ahead was that the spot of drugs on the plate was too tenuous, and the Raman signal was too weak, thus, we focused on the concentration of the spot on TLC plate, and aimed to develop a new method to deal with the spots of six glucocorticoids. The objective of this study was to screen for the presence of six glucocorticoids added to dietary supplements by TLC-SCRS.

## 2. Results and Discussion

### 2.1. Thin-Layer Chromatography Condition

The six reference substance solutions and mixture solution were spotted on one TLC plate and then were eluted in a developing chamber with the optimum mobile phase. After the elution, the TLC plate was evaporated naturally, and the separated spots were then visualized and located under ultraviolet illumination at 254 nm.

**Figure 1:**
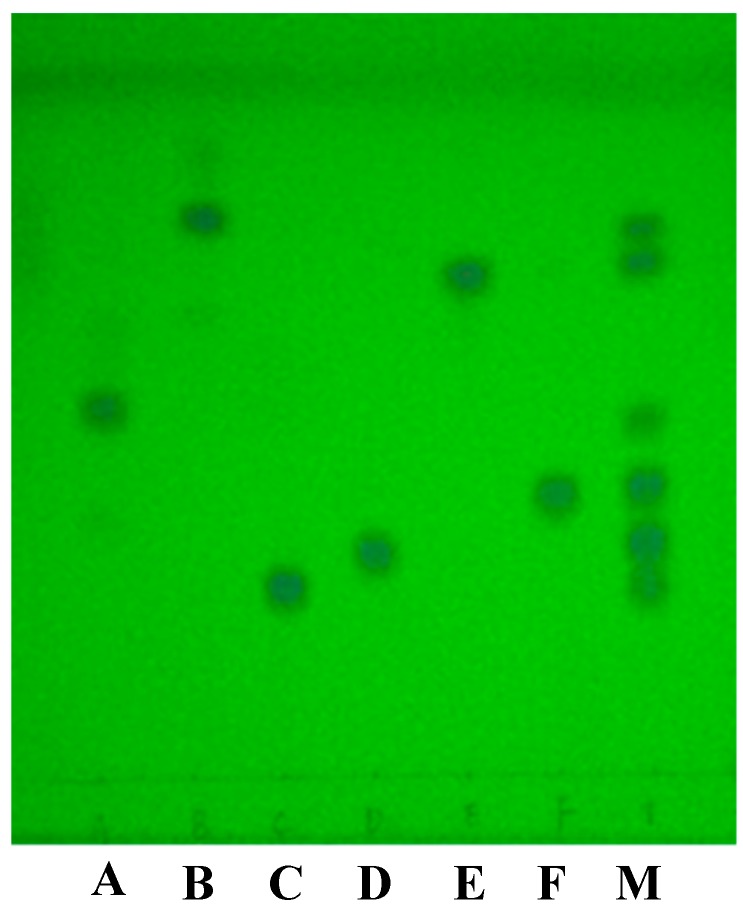
Results from TLC analysis of the six reference chemicals and mixture solution. A, B, C, D, E, F: reference substance of prednisone, prednisone acetate, prednisolone, hydrocortisone, hydrocortisone acetate, and dexamethasone; and M: mixture solution.

Since the polarity of the six analytes did not differ substantially, dissimilar developing solvents prepared from a variety of solvents were investigated to achieve complete separation of the six analytes. Through the investigation and optimization of several different developing solvents [26] by detecting the spots under 254 nm, when dichloromethane-acetone-methanol = 12:2:0.5, (*v*/*v*/*v*) was used as the optimum mobile phase, the six analytes were mutually separated (Figure 1).

The R_f_ value of prednisone, prednisone acetate, prednisolone, hydrocortisone, hydrocortisone acetate, and dexamethasone on the TLC plate were 0.44, 0.75, 0.25, 0.28, 0.71, 0.35, respectively. Due to the similar structure, some of them had similar R_f_ values, such as prednisolone and hydrocortisone or prednisone acetate and hydrocortisone acetate. Thus, we could not define the spot only by the R_f_ value. Combining the Raman spectra of spots to distinguish the spots having similar R_f_ value, could enhance experimental specificity.

The R_f_ value was calculated as follows:

(1) $$R_{f}=\frac{\mathrm{distance}\;\mathrm{of}\;\mathrm{the}\;\mathrm{substance}\;\mathrm{zone}\;\mathrm{from}\;\mathrm{the}\;\mathrm{sample}\;\mathrm{origin}\;(\mathrm{mm})}{\mathrm{solvent}\;\mathrm{front}\;\mathrm{migration}\;\mathrm{distance}\;(\mathrm{mm})}$$

Note: The distance was measured from the application line to the middle of the substance spots. Spots in the mixture solution should have similar R_f_ values, shape, and color as spots derived from the standard compounds.

The deposition amount on TLC plate was calculated as follows:

Deposition amount (μg) = Concentration (μg/μL) × Volume of spotting (μL)(2)

### 2.2. The TLC-SCRS Method

In this method, a great challenge ahead was the drug spot on the plate usually could not meet the demand of Raman spectroscopy detection sensitivity; we focused on the drug spot concentration on the TLC plate, and aimed to develop an effective method to obtain Raman signals of the six glucocorticoids. 

**Figure 2:**
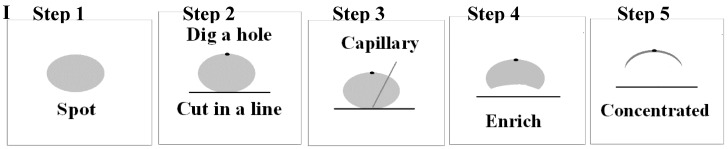

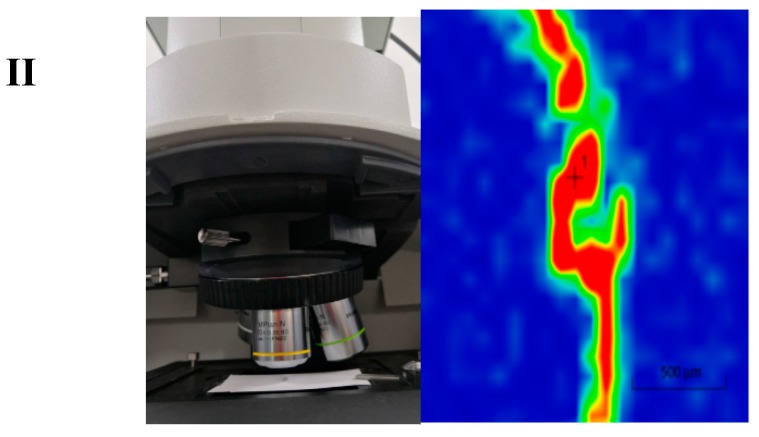
The detailed process of spot concentration on TLC plate (**I**), and micro-Raman imaging map (**II**).

Sample solutions (the diameters were no more than 4 mm) were spotted on one TLC plate and eluted in a developing chamber with dichloromethane-acetone-methanol (12:2:0.5). After the elution, the TLC plate was evaporated naturally, the separated spots were then visualized and located under ultraviolet illumination at 254 nm, and after that we concentrated the drug spot on the TLC plate, following the steps with ethanol (shown in Figure 2I). Finally, the concentrated spot was obtained on which we collected Raman signals.

The detailed process of spot concentration on TLC plate was as follows: when we look down at the plate after location under 254 nm ultraviolet illumination, we could see the spot on the plate (step 1); then we cut the bottom edge of the spot with a knife in a line that is tangent to the spot, and dug a small hole on the opposite edge of the spot to the depth that aluminum plate could be seen (step 2); then 10 μL ethanol was spotted on the point of tangency with a capillary (step 3); the drug in the spot was eluted from the bottom edge to the top edge (step 4); Raman signals were collected on the drug-concentrated parts (step 5); finally, the concentrated spot on the TLC plate was detected under the laser, and the micro-Raman imaging map was obtained (Figure 2II).

### 2.3. Comparation with TLC-SERS Method

**Figure 3:**
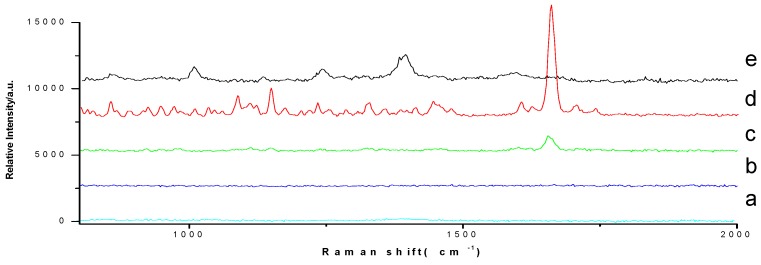
Raman spectra of prednisone acetate (**a**: detected by TLC-SERS method without prednisone acetate, **b**: detected directly on TLC spot without prednisone acetate, **c**: detected by the TLC-SCRS method, **d**: detected directly on reference powder, and **e**: detected by the TLC-SERS method. The deposition amount is 10 μg).

**Figure 4:**
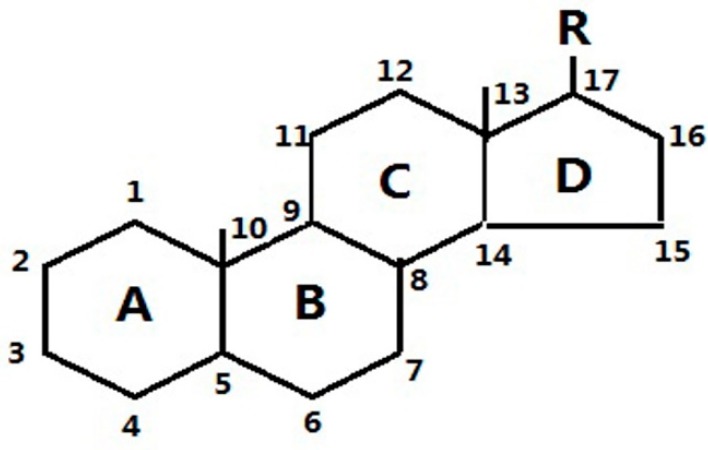
The common structure of steroids.

TLC-SERS method had been widely used in Raman signal enhancement. In order to compare the two methods, TLC-SERS method was used to enhance Raman signal on TLC plate, in which silver colloids were prepared following the conventional heating method studied by Lee and Meisel [27]. Taking prednisone acetate as an example, Raman spectra by the TLC-SERS method was compared with TLC–SCRS method (Figure 3). We did not see any peak on the TLC spot directly with the deposition amount of 10 μg (Figure 3b), while a strong peak at 1658 cm^−1^ was detected when the TLC-SCRS method was used (Figure 3c), which was at the same Raman shift with the reference powder (Figure 3d). At the same time, the other peaks were too small to be seen in the spectra by the TLC-SCRS method compared with the spectra of the reference powder. The reason considered was that the signal value which was associated with the deposition amount was too small to exceed the noise value. When the TLC–SERS method was used, totally different Raman shift peaks were detected. The Raman spectra were detected on the TLC plate with silver colloids, and no peak was found (Figure 3a). When the TLC–SERS method was used for the prednisone acetate spot on TLC plate, the peaks that arose from δ_as_(CH_2_), δ(CH) (1395 cm^−1^) and δ(CH), δ(CC)_ringD_ (Figure 4) (1012 cm^−1^) [28] were strengthened, and there was almost no peak in the position of prednisone acetate’s characteristic peak (1660 cm^−1^) (Figure 3e).

Comparing with TLC–SERS method, TLC-SCRS method had similar Raman shift with the reference powder, although the signal intensity was weaker. Furthermore, reference substances were not necessary in the TLC-SCRS method, as the Raman signal could be compared with the standard atlas, directly. From this point, the TLC-SCRS method was more convenient.

Furthermore, since the target reagent on TLC sometimes cannot be observed by SERS, which has selectivity via the binding affinity on the metal surface, the TLC-SCRS method is important.

### 2.4. Raman Spectra by TLC–SCRS

**Figure 5:**
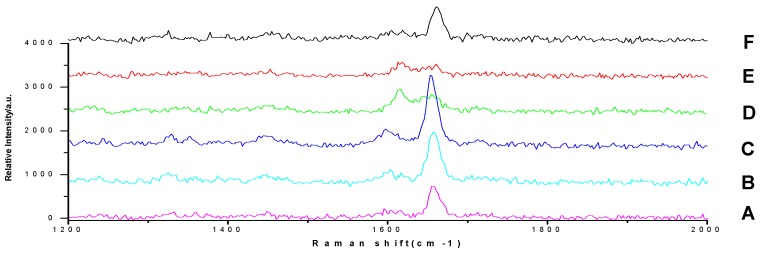
Raman spectra of reference substance solutions by TLC–SCRS (deposition amount of 10 μg). **A**, **B**, **C**, **D**, **E**, **F**: prednisone, prednisone acetate, prednisolone, hydrocortisone, hydrocortisone acetate, and dexamethasone.

Reference substance solutions were analyzed by use of this TLC–SCRS method by acquiring the spots spectra (Figure 5) on TLC plate in Figure 1. 

Raman spectra of reference substance prednisone, prednisone acetate, prednisolone, hydrocortisone, hydrocortisone acetate, and dexamethasone were detected directly on reference substance powders, respectively (Figure S1).

As we can see in Figure 5 and Figure S1, and Table 1, the Raman shift on these spectra by TLC–SCRS method were basically the same as those obtained from reference chemicals, indicating that we could obtain the spectral character by the TLC–SCRS method, although there were differences in the peak height, peak separation, and shape.

The six glucocorticoids are all steroid hormone drugs and have the same structure of cyclopentanoperhydrophenanthrene (Figure 4). Ring A in the structure has the cyclohexene structure, which causes two strong Raman signals. The signal of rings B, C, and D were weak, so the characteristic peaks all came from ring A.

The Raman scattering characteristic peak of hydrocortisone and hydrocortisone acetate were similar (Table 1), and there were double strong peaks between 1652 cm^−1^ and 1613 cm^−1^, both coming from the cyclohexene structure, and peak separations were both less than 40 cm^−1^, so it was difficult to identify hydrocortisone and hydrocortisone acetate only by their Raman spectra. The Raman scattering characteristic peaks of prednisone, prednisone acetate, prednisolone, and dexamethasone acetate were similar, and there were a strong peaks and a weak peaks, both coming from the cyclohexadiene structure. There was still a strong peak between 1655 cm^−1^ and 1661 cm^−1^, but due to the influence of 1,2-cyclohexadiene, the signal between 1598 cm^−1^ and 1603 cm^−1^ was attenuated to a weak peak. Additionally, peak separations were all more than 50 cm^−1^. It was also difficult to identify prednisone, prednisone acetate, prednisolone, and dexamethasone only by their Raman spectra. Thus, there were distinct differences between cyclohexene and cyclohexadiene steroid hormone drug structures in terms of their Raman spectra. Only the two types of compounds could be identified by Raman spectroscopy.

In summary, the combination of the R_f_ value on the TLC plate and Raman scattering characteristic peaks in the Raman spectra showed complementary advantages, and the specificity of the identification method could be effectively enhanced. For example, the R_f_ values of prednisolone and hydrocortisone were similar, but there were significant differences in their Raman scattering characteristic peaks. The Raman scattering characteristic peaks of prednisone, prednisone acetate, prednisolone, and dexamethasone were similar, but there were significant differences in the R_f_ values on the TLC plate. Thus, the specificity of identification was enhanced by the TLC–SCRS method to achieve the purpose of accurate identification.

### 2.5. Analysis of Simulated Positive Samples

Five kinds of dietary supplements (Table S1) were taken as the negative samples (which were established as not containing prednisone, prednisone acetate, prednisolone, hydrocortisone, hydrocortisone acetate, and dexamethasone by Qiqihar Institute for Food and Drug Control). Five kinds of dietary supplement simulated positive samples were prepared by taking the single oral dose of glucocorticoids as the minimum necessary amount of adulterant in one dosage. Negative sample solution and dietary supplement simulated positive solutions were prepared in accordance with the preparation method of real dietary supplement solutions. The six glucocorticoids in dietary supplement simulated positive samples were detected, respectively, paying close attention to the influence of those matrix compositions in dietary supplement simulated positive samples at the same time. The results showed that the five kinds of substrates in dietary supplements had no interference on the Raman spectra.

Taking dietary supplement 1 as an example of a negative sample, a simulated positive sample solution was prepared (Table S2), and the TLC plate (Figure S2) was obtained by the TLC-SCRS method. The simulated positive sample solution showed spots at the same location with reference substance solutions (A, B, C, D, E, F), respectively. However, there were no spots in the simulated negative samples observed, indicating that the matrix of sample 1 had no interference on the TLC analysis of the six glucocorticoids.

**Figure 6:**
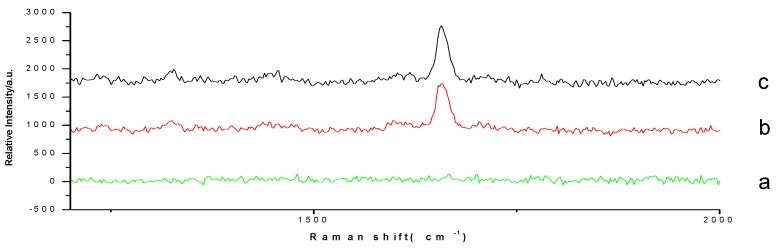
TLC-SCRS of prednisone (A) (**a**: simulated negative sample; **b**: simulated positive sample; and **c**: reference substance of prednisone).

Raman spectra of prednisone (A) reference substances and the prednisone spot in the simulated positive sample were detected (Figure 6). The results showed that the prednisone reference substance and the simulated positive samples had the same Raman scattering characteristic peak, and there was no signal for negative samples. This indicated that the matrix of dietary supplement 1 had no interference with prednisone’s in situ enrichment Raman spectrum.

In the same way, the Raman spectra of the other reference substances and simulated positive samples on TLC plates were detected, respectively (Figure S3), the results showed that the other reference substances and the simulated positive samples had the same Raman scattering characteristic peak, and there was no signal for negative samples. It was indicated that the matrix of dietary supplement 1 had no interference with TLC on-site spot-concentrated Raman spectra of prednisone acetate, prednisolone, hydrocortisone, hydrocortisone acetate, or dexamethasone.

### 2.6. Inspection of Limit of Detection (LOD)

**Figure 7:**
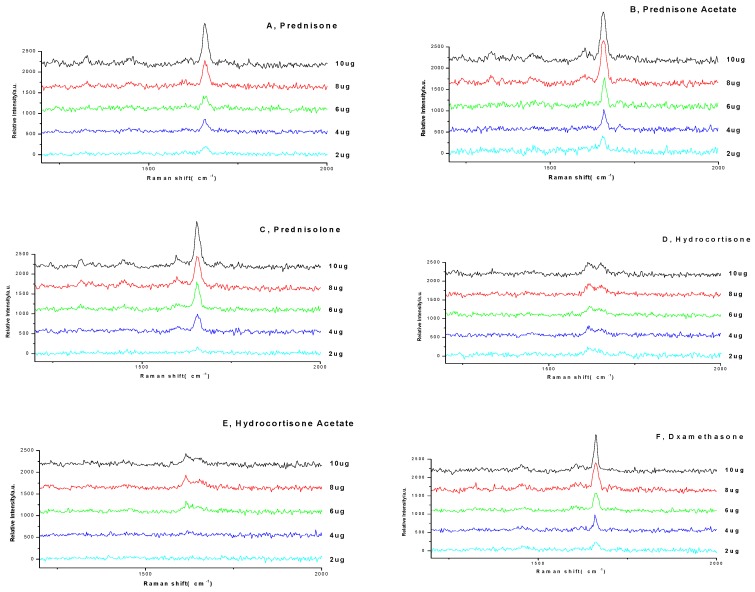
The Raman spectra of different deposition amount of reference substances on TLC.

Reference substance solutions (prednisone, prednisone acetate, prednisolone, hydrocortisone, hydrocortisone acetate, and dexamethasone) were spotted on the TLC plate separately, and the drug deposition amount of each spot was 2–10 μg, respectively. The spectra by TCL-SCRS were recorded in Figure 7.

**Figure 8:**
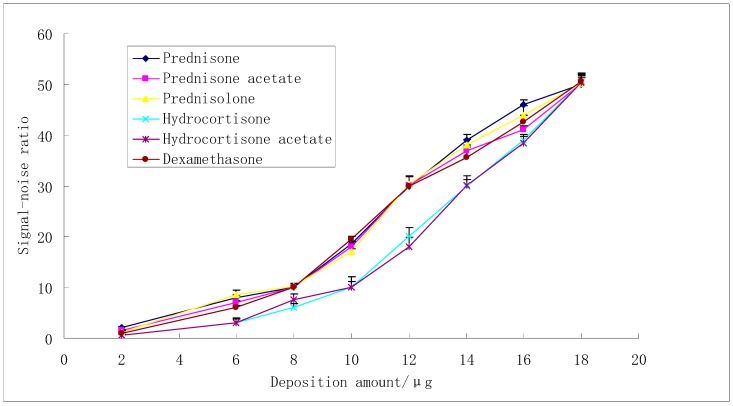
The LOD analysis of six reference substances.

The LOD analysis of six reference substances was shown in Figure 8. When S/N was 3, the deposition amount of the corresponding reference substances (ug) was the limit of detection (LOD). The LOD of reference substances (A, B, C, D, E, and F) were 4 μg, 4 μg, 4 μg, 6 μg, 6 μg, and 4 μg, lower than the deposition amounts which were related with the minimum necessary amount of adulterants for drug use (Table S2). Thus, the TLC-SCRS method could be used to detect glucocorticoids illegally added to dietary supplements. When S/N was more than 8, the signal noise ratio was linear to the deposition amount of the corresponding reference substances (ug), and the linear equations were y(A) = 4.45x − 24.93 (r = 0.9970), y(B) = 3.94x − 20.19 (r = 0.9909), y(C) = 4.13x − 22.03 (r = 0.9908), y(D) = 4.55x − 33.27 (r = 0.9937), y(E) = 4.34x − 30.29 (r = 0.9919), and y(F) = 3.96x − 20.11 (r = 0.9954). The recoveries of six glucocorticoids in simulated positive samples were 95.6%, 92.3%, 93.5%, 96.1%, 92.7%, and 94.6%, respectively.

### 2.7. Detection of Real Samples

Based on the research above, the R_f_ value on the TLC and the characteristic peaks in the Raman spectra related to the six glucocorticoids had been found, and it was reasonable to take them as the monitoring index for further investigation of adulteration in dietary supplements. In order to further confirm the performance of TLC-SCRS, it was employed to detect five real samples provided by the Qiqihar Institute for Food and Drug Control, including decoction, powder, capsule, and tablet.

**Figure 9:**
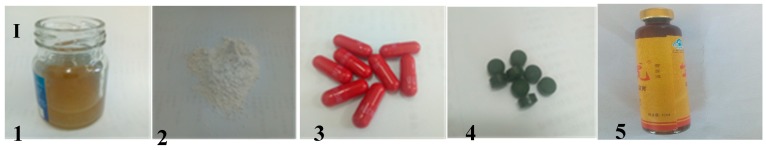

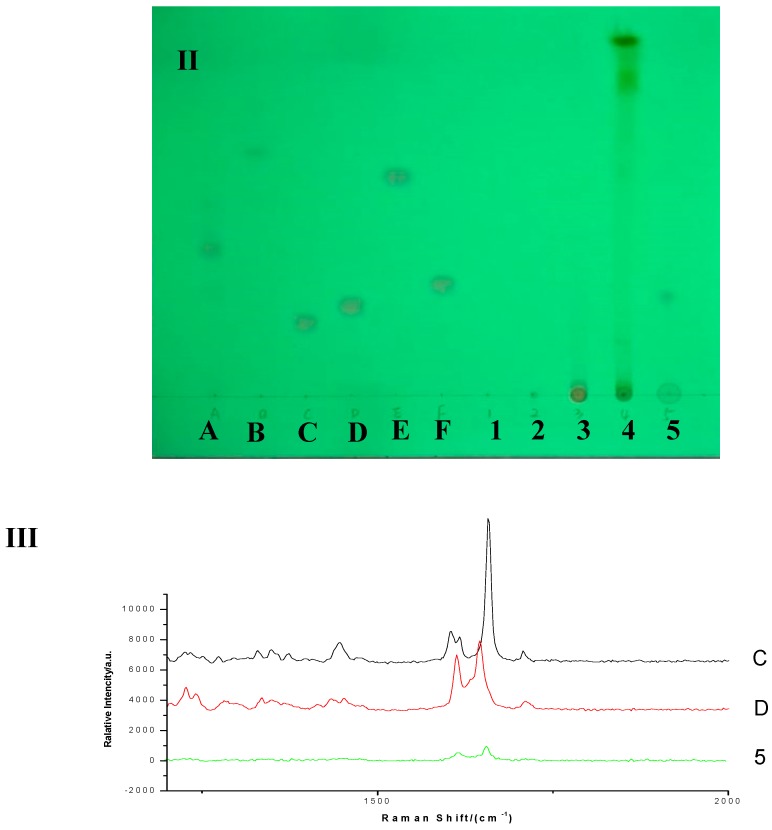
Five real samples used in TLC-SCRS detection (**I**), results from TLC analysis of five real samples developed with dichloromethane-acetone-methanol 12:2:0.5 (*v*/*v*/*v*) (**II**), (A–F: prednisone, prednisone acetate, prednisolone, hydrocortisone, hydrocortisone acetate, and dexamethasone references 1–5: sample 1–5), and the Raman spectra obtained from prednisolone, hydrocortisone, and sample 5 (**III**).

There was no spot detected in samples 1, 2, 3, and 4, indicating there were no adulterants (prednisone, prednisone acetate, prednisolone, hydrocortisone, hydrocortisone acetate, and dexamethasone) in the four samples. There was only a spot in sample 5 (R_f_ = 0.27) that appeared at almost the same R_f_ value with prednisolone (R_f_ = 0.25) or hydrocortisone (R_f_ = 0.28). We obtained the spectra of the spot in sample 5 after careful inspection, which was compared with the spectra of prednisolone and hydrocortisone references. As shown in Figure 9, the Raman spectra indicating the characteristic peaks of hydrocortisone were detected in sample 5. There were no illegal ingredients mentioned above in samples 1, 2, 3, and 4, but hydrocortisone was found in sample 5.

### 2.8. HPLC-MS Verification

To further confirm the TLC-SCRS results, all the real samples were also analyzed by HPLC-MS, and the same results were obtained (Figure S4). There were no illegal ingredients in samples 1, 2, 3, and 4. However, hydrocortisone was found in sample 5. Although TLC-SCRS was less sensitive than HPLC-MS, the six glucocorticoids could be accurately and quickly detected by use of the TLC-SCRS method at effective doses, which were above the LOD of the TLC-SCRS.

## 3. Experimental

### 3.1. Materials and Reagents

TLC aluminum plates (Merck KGaA, Darmstadt, Germany) consisted of high-performance silica gel 60 F_254_ plates, the silica gel particle size was 8 ± 2 μm, and the layer thickness was 0.2 ± 0.03 mm. There was a fluorescing additive, F_254_, on the plates used for the visualization of the spot. An invisible spot in the sunlight could be seen under UV light of 254 nm because of the fluorescent indicators.

The six reference substances (prednisone, prednisone acetate, prednisolone, hydrocortisone, hydrocortisone acetate, and dexamethasone) were purchased from the National Institutes for Food and Drug Control (Beijing, China), and were diluted to the desired concentrations by using analytical-grade Anhydrous ethanol was purchased from Tianjin Tianli Chemical Reagent Co. Ltd. (Tianjin, China).

### 3.2. Apparatus

Separated spots were located under 254 nm by use of an ultraviolet analyzer (YOKO-2F; Wuhan YOKO Technology Ltd., Wuhan, China). Spot absorption spectra were obtained by use of a DXR™ xi Raman Imaging Microscope (Thermo Fisher Scientific, Waltham, MA, USA) with an excitation wavelength of 780 nm, a resolution of 5 cm^−1^ and a 10× long working distance microscope objective. The excitation power was 24 mW, the integration time was 0.2 s, and the number of scans was 20. The scan range was 100–3300 cm^−1^, and a 50 µm confocal pinhole DXR780 full range grating (400 line/mm) was used. The detector was a TE-cooled electron-multiplying CCD (EMCCD).

### 3.3. Sample Preparation

Reference substance solutions (prednisone, prednisone acetate, prednisolone, hydrocortisone, hydrocortisone acetate, and dexamethasone) were prepared by dissolving each reference substance in ethanol ultrasonically at a concentration of 1 mg/mL for 10 min, separately. 

Mixture solutions (prednisone, prednisone acetate, prednisolone, hydrocortisone, hydrocortisone acetate, and dexamethasone) were prepared by dissolving every reference substance in ethanol ultrasonically at a concentration of 1 mg/mL for 10 min.

Simulated negative sample solutions were prepared by mixing single dosage negative sample drugs (which were established to not contain prednisone, prednisone acetate, prednisolone, hydrocortisone, hydrocortisone acetate, and dexamethasone by Qiqihar Institute for Food and Drug Control) with 5 mL ethanol, treating with ultrasound for 10 min, and then filtrating with a microfiltration membrane. 

Simulated positive sample solutions were prepared by mixing six reference substances (prednisone, prednisone acetate, prednisolone, hydrocortisone, hydrocortisone acetate, and dexamethasone) with the simulated negative sample solutions at a concentration of 1 mg/mL, treating with ultrasound for 10 min, and then filtrating with a microfiltration membrane.

Real sample solutions were prepared by grinding or dissolving real dietary supplements and then extracting ultrasonically with ethanol, and then filtrating with microfiltration membrane.

## 4. Conclusions

In summary, a novel combined technique of TLC and SCRS was established to separate and detect small amounts of the six glucocorticoids added to dietary supplements for the first time. Additionally, the developed TLC-SCRS method showed enough sensitivity, specificity, and stability for the ingredients we detected. The optimum SCRS experimental conditions were found. Real samples were then analyzed by use of the TLC-SCRS method and the results were consistent with that obtained by the conventional method of HPLC-MS. The newly-developed TLC-SCRS method without reference substances was much simpler and faster for the detection of adulterants in real dietary supplements than classical methods. We hope the new method may provide more opportunities for the detection of illegal adulterants.

## Figures and Tables

**Table 1:** Raman spectral characteristic peaks of the six reference substances.

Adulterants	Structure	Raman Shift (cm^−1^) of Spot on TLC	Raman Shift (cm^−1^) of Reference Powder	Intensity
Prednisone (A)	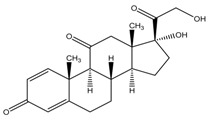	1657	1658	s
		1602	1607	w
Prednisone acetate (B)	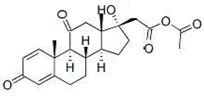	1658	1660	s
		1603	1606	w
Prednisolone (C)	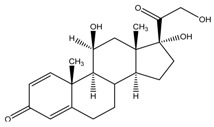	1655	1658	s
		1605	1604	w
Hydrocortisone (D)	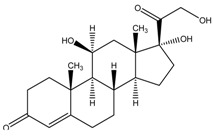	1652	1645	s
		1614	1613	s
Hydrocortisone acetate (E)	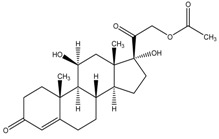	1628	1629	s
		1617	1619	s
Dexamethasone (F)	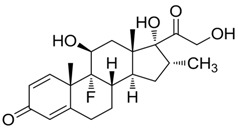	1661	1658	s
		1605	1606	w

s, strong; w, weak.

## Supplementary Materials

The following are available online, Figure S1: Raman spectra of reference substance powders, Figure S2: TLC of the simulated positive sample, Figure S3: TLC on-site spot concentrated Raman spectra of the simulated positive sample, Figure S4: HPLC-MS results of sample 5, Table S1: The main drug matrix of 5 kinds of dietary supplement, Table S2: The preparation of simulated positive sample solutions.
