# Supplementary material for: Rapid Detection of Six Glucocorticoids Added Illegally to Dietary Supplements by Combining TLC with Spot-Concentrated Raman Scattering
Source: Molecules. 2018 Jun 21;23(7):1504. doi: 10.3390/molecules23071504 (PMC6100086; doi:10.3390/molecules23071504)
Supplement: Supplementary file 1 [file molecules-23-01504-s001.pdf]

molecules

MDPI

Article

Rapid Detection of Six Glucocorticoids Added Illegally to Dietary Supplements by Combining TLC with Spot-Concentrated Raman Scattering

Li Li, Xin Liang *, Tao Xu, Feng Xu and Wei Dong

College of Pharmacy, Qiqihar Medical University, Qiqihar 161006, China; lilianlinsuo@163.com (L.L.); Xutao@qmu.edu.cn (T.X.); 15845205504@163.com (F.X.); pingguoweiweiwei@126.com (W.D.)

* Correspondence: liangxin@qmu.edu.cn; Tel.: +86-0452-2663172

Supplementary Data

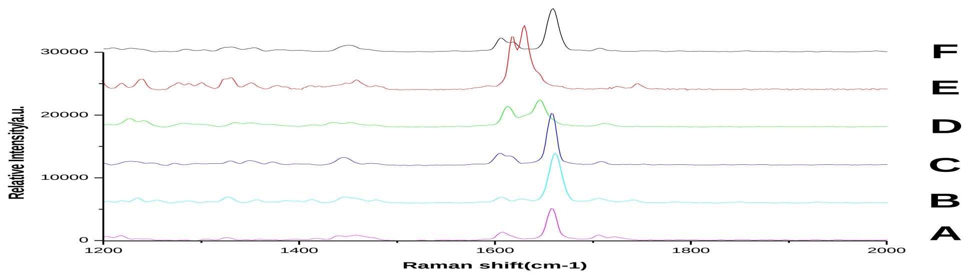

Figure S1 Raman spectra of reference substance powders (A, B, C, D, E, F: prednisone, prednisone acetate, prednisolone, hydrocortisone, hydrocortisone acetate, and dexamethasone)

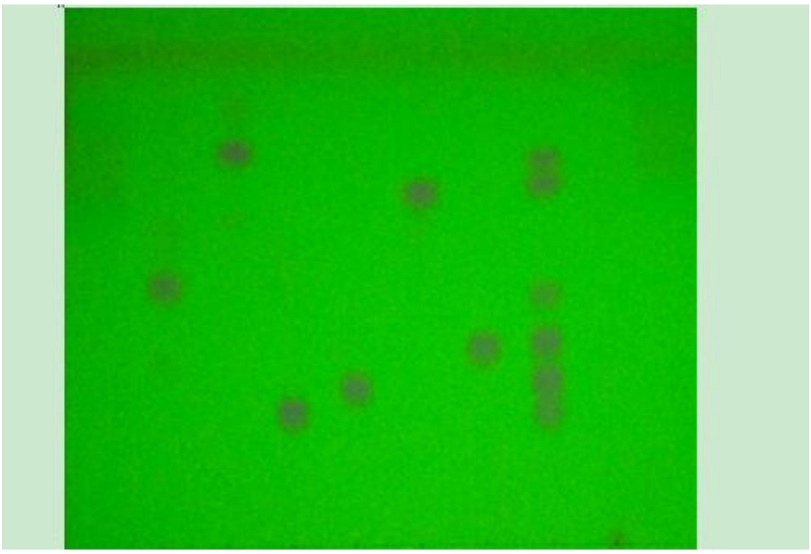

A B C D E F + −

Figure S2. TLC of the simulated positive sample (A, B, C, D, E, F: Reference substance of prednisone, prednisone acetate, prednisolone, hydrocortisone, hydrocortisone acetate, dexamethasone; +: Simulated positive sample; -: Simulated negative sample)

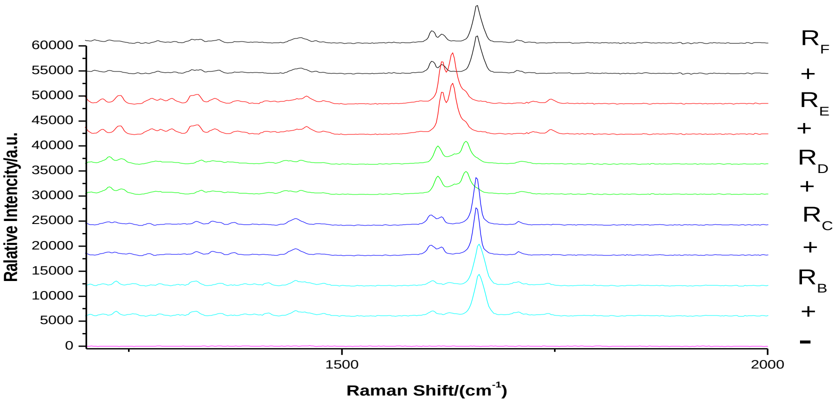

Figure S3. *TLC on-site spot concentrated Raman spectra* of the simulated positive sample (R_B, R_C, R_D, R_E, R_F: Reference substance of prednisone acetate, prednisolone, hydrocortisone, hydrocortisone acetate, dexamethasone; +: Simulated positive sample; -: negative sample;)

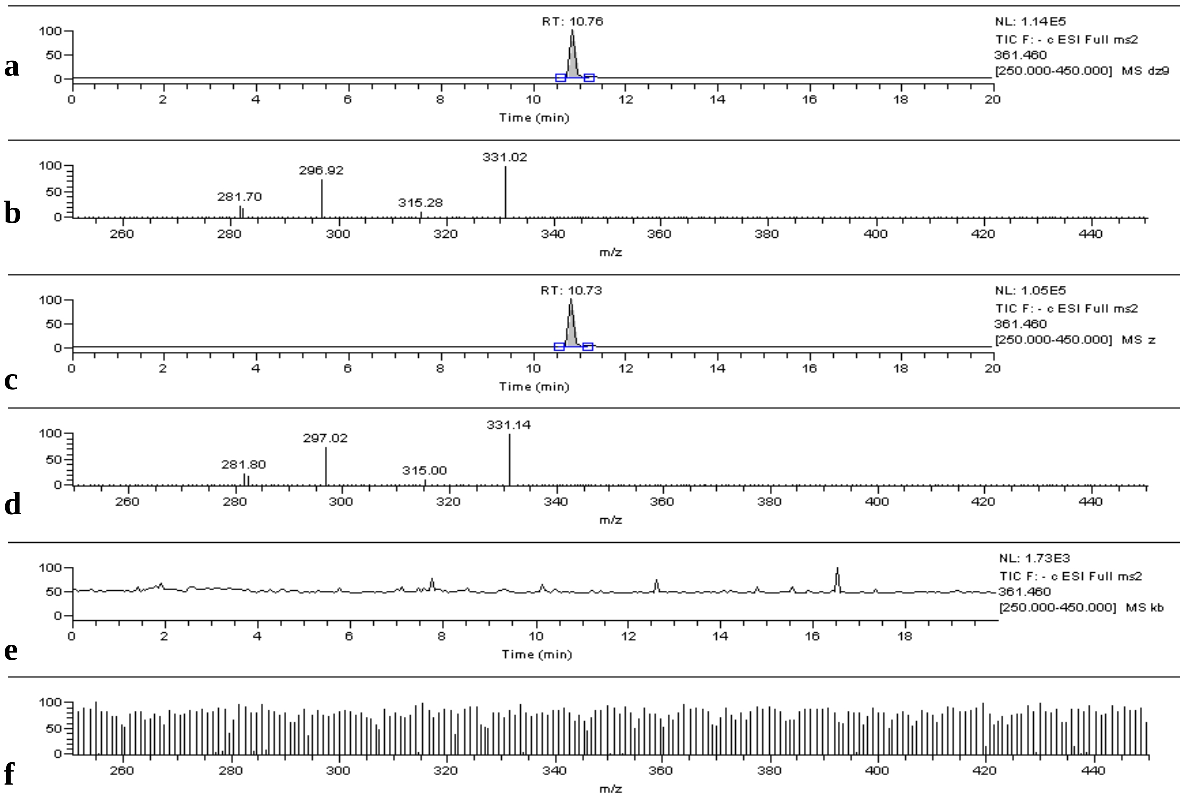

Figure S4. HPLC-MS results of sample 5 (a: ion chromatogram of hydrocortisone reference, b: mess spectrogram of hydrocortisone reference, c: ion chromatogram of sample 5, b: mess spectrogram of sample 5, a: ion chromatogram of blank control, b: mess spectrogram of blank control)

Table S1. The main drug matrix of 5 kinds of dietary supplement

Dietary supplements	Lot.	Dose(g)	Main drug matrix
1	5205003	5	*Clausinella tiara*、*Jujube*、*Zingiber officinale Roscoe*、*Rock candy/crystal sugar*
2	20151020	2	*Dioscorea opposita Thunb.*、*Calcium Gluconate*、*Calcium Ascorbate*、*Biological calcium*、*Taurine*、*Papain*、*Stevia sugar*
3	20160301D	1.05	*Panax quinquefolius L.*、*Rhodiola rosea L.*、*Gynostemma pentaphyllum Makino*、*Lycium chinenseMill.*、*Asini Corii Colla*、*Cornu Cervi Pantotrichum*
4	617010201	2	*Spirulina*
5	20170505	0.88	*Amino acid*、*Taurine*、*Cephalin*、*Deep-sea animal extracting solution*、*Dimocarpus longgana Lour.*、*Common Yam Rhizome*、*Lycium chinenseMill.*、*Jujube*

Table S2. The preparation of simulated positive sample solutions

Adulterants	Minimum necessary amount of adulterant for a drug use	Concentration	Volume	Deposition amount	*LOD*
Prednisone	5 mg	2 mg/1 mL	6 µL	12 µg	4 µg
Prednisone acetate	5 mg	2 mg /1 mL	6 µL	12 µg	4 µg
Prednisolone	5 mg	2 mg /1 mL	6 µL	12 µg	4 µg
Hydrocortisone	10 mg	2 mg /1 mL	6 µL	12 µg	6µg
Hydrocortisone acetate	20 mg	2 mg /1 mL	6 µL	12 µg	6 µg
Dexamethasone	0.75 mg	0.75 mg /1mL	6 µL	4.5µg	4 µg
